# The Association Between Breastfeeding Duration and Attentional and Cognitive Development in Infancy and Early Childhood

**DOI:** 10.1111/infa.70120

**Published:** 2026-08-19

**Authors:** Feline A. van Gelderen, Roy S. Hessels, Kelly A. Mulder, Lidewij Schipper

**Affiliations:** ^1^ Danone Research & Innovation Utrecht the Netherlands; ^2^ Experimental Psychology Helmholtz Institute, Utrecht University Utrecht the Netherlands

**Keywords:** attentional disengagement, breastfeeding, early‐life nutrition, gap‐overlap paradigm, infancy

## Abstract

While breastfeeding duration has been positively associated with later cognitive outcomes in childhood, behavioral assessments commonly used in early infancy may lack the sensitivity required to detect subtle differences. This study examined whether eye‐tracking during infancy can be used to detect differences in attentional development related to infant feeding practices in a generally well‐nourished population. Specifically, using data from the YOUth Baby & Child cohort (*N* = 2647) collected in Utrecht, the Netherlands, infant attentional disengagement was assessed around 5 months (4–6 months) and around 10 months (9–11 months) of age, alongside child receptive vocabulary around 3 years (2–4 years) and around 6 years (5–7 years) of age. While a positive association was established between breastfeeding duration and receptive vocabulary around 6 years of age, no association was established between breastfeeding duration and attentional disengagement at either timepoint. These findings suggest that effects of early‐life nutrition on cognitive development in well‐nourished infants do not extend to early attentional development or are too subtle to be picked up using eye‐tracking. Future research could explore populations with greater developmental variability.

## 1 Introduction

Human milk supports optimal health, growth and development throughout infancy (Lessen and Kavanagh 2015; Meek and Noble 2022), which is why the WHO recommends exclusive breastfeeding for the first 6 months and continued breastfeeding with complementary foods up to 2 years or beyond (World Health Organization and United Nations Children’s Fund 2003). For infants who do not receive human milk, commercially available infant formulas serve as the preferred substitute (Martin et al. 2016). Infant formula is designed to mimic human milk as closely as possible, yet human milk is not fully replicable through industrial production—at least in part due to its complex and dynamic nature (Talbert and Townsend 2025). Accordingly, disparities in both short‐ and long‐term health outcomes are observed when comparing breastfed to formula‐fed infants. While it must be noted that most studies in the field are observational, breastfed infants show reduced risk of morbidity (Masi and Stewart 2024), different growth patterns (Butte et al. 2000; Zong et al. 2020), improved structural brain development (Chade et al. 2024; Deoni et al. 2018) and a more diverse gut microbial profile (Inchingolo et al. 2024).

Exclusive and prolonged breastfeeding is also associated with improved scores of children and early adolescents on tests of overall intellectual ability and tests pertaining to a specific cognitive domain such as language (Horta et al. 2015; Lovcevic 2023). Although these associations are usually attenuated and in some studies disappear after adjustment for confounders (Walfisch et al. 2013), there is also evidence supporting a causal effect (Kramer et al. 2008). Screening tools relying on direct observation, parent‐report or combinations thereof, such as the Bayley Scales of Infant Development (Bayley 2006), remain the dominant means of cognitive assessment for infants and young children (for an overview, see Isaacs et al. 2008). The use of such tests has allowed for differences between formula‐fed and breastfed infants to be distinguished from the first year of life onwards (Boutwell et al. 2012; Lee et al. 2016). It is however thought that behavioral assessments may lack sensitivity when used for younger infants (McCann et al. 2020), which has been shown previously in the context of detecting outcomes related to prematurity (Kaltsa et al. 2024). Early detection of how nutrition affects child neurocognitive development could enable the timely assessment of potential risk as well as the success of nutrition interventions.

To address the limited sensitivity of behavioral measures in early infancy, researchers may turn to alternative methods that can be applied at younger ages, including neuroimaging and eye‐tracking techniques. The use of neuroimaging techniques such as electroencephalography (EEG) and magnetic resonance imaging (MRI) in infant populations has allowed for researchers to identify differences between formula‐fed and breastfed infants within the first 6 months of life (Gilbreath et al. 2024). Whilst providing new insights, many neuroimaging techniques come with the drawback of being more costly, both in terms of equipment and required training for personnel to operate it. Constraints of a more practical nature may apply too, like the demand for a degree of immobility (Isaacs 2013). Given these practical limitations, eye‐tracking offers a promising alternative for early assessment of neurocognitive development. By virtue of its non‐invasive nature and minimal participant requirements, there are no inherent age restrictions: eye‐tracking may even be performed on newborns (e.g., Macchi Cassia et al. 2001; Papageorgiou et al. 2015). It is also very suitable for mapping developmental trajectories, as the same test can be used with participants of all ages. Though infant eye‐tracking studies go back decades, technological advances as well as establishment of experimental paradigms have expanded their applicability across research fields (Gredebäck et al. 2009; Hessels and Hooge 2019; Oakes 2012). The utility of eye‐tracking in studying effects of the early‐life nutritional environment on neurocognitive development of infants has received increasing support of late. However, its application has so far been largely restricted to populations that may be at risk of neurodevelopmental delay due to malnourishment or specific nutrient deficiencies (Leppänen et al. 2022; McCann et al. 2023; Prado et al. 2020). This raises the question of whether eye‐tracking may also be suitable for detecting more subtle differences in cognitive development, such as those associated with breastfeeding, among typically well‐nourished infants.

The gap‐overlap paradigm, which has been used extensively to study attentional disengagement and its development, is particularly suitable because it is directly applicable across all ages and its psychometric properties have been evaluated (Cousijn et al. 2017). Attentional disengagement may be understood as the step that necessarily succeeds the engagement of visual attention at a particular stimulus and precedes the shifting of visual attention from this first stimulus to another (Colombo 2001; Posner et al. 1987). The ability to disengage visual attention is thought to be important to learning in general and self‐regulation in particular (see for a review: Rothbart et al. 2011). Measures of the gap‐overlap paradigm have been previously—though not consistently—brought into association with later developmental outcomes, specifically executive attention (e.g., Hitzert et al. 2014) and diagnosis of autism spectrum disorder (Elsabbagh et al. 2013; Keehn et al. 2021, 2024; but see J. Fischer et al. 2016). In the gap‐overlap paradigm, participants are presented with a central stimulus that acts as a fixation point and a subsequent peripheral stimulus that is meant to elicit a rapid eye movement (i.e., saccade) toward it (Hendry et al. 2019; Saslow 1967). The saccadic reaction time (SRT) is recorded, formally “the time interval between an event taking place and the first recorded oculomotor response to that event” (Wass et al. 2014). Two conditions are employed: the central stimulus either remains on‐screen (*overlap*) or disappears shortly before the peripheral stimulus appears (*gap*). The typical outcome is that SRTs (measured from peripheral stimulus onset) are shorter in gap trials (Abrams et al. 1998; B. Fischer and Weber 1993), a robust phenomenon coined the “gap effect” (Saslow 1967). It is key to distinguish that the gap condition facilitates early disengagement (upon disappearance of the central fixation point), whereas in the overlap condition active disengagement from the central fixation point is required and initiated only upon peripheral target onset. Thus, the difference in SRT between the gap and overlap condition is accounted for by the additional time it takes to disengage included in the overlap trials. The size of the gap effect is therefore interpreted as an inverse estimate of attentional disengagement efficiency.

It is known that young infants are not readily capable of looking away once their attention has been engaged, showing profound disengagement difficulty referred to as “sticky fixation” (Hood 1995) or “obligatory looking” (Stechler and Latz 1966). The ability to disengage attention undergoes major change within the first months after birth (Colombo 2001) and becomes evident around 4 months of age (Frick et al. 1999; Johnson et al. 1991; Matsuzawa and Shimojo 1997; McConnell and Bryson 2005). It has also been established that the gap effect is larger in children (worse performance) than in adults (Cohen and Ross 1977) and more recently a decrease in size throughout adolescence was demonstrated (Van der Stigchel et al. 2017). In one of the previous studies that utilized eye‐tracking to assess cognitive development during infancy, the gap effect was shown to be sensitive to infant iron status within a sample of iron‐sufficient and iron‐deficient infants (McCann et al. 2023).

To investigate whether measures derived from the gap‐overlap paradigm might be sensitive to early nutrition exposure in a population of infants assumed to be generally well nourished, we leveraged data collected as part of the broad‐scope Youth Of Utrecht (YOUth) cohort study, which included not only scores on standardized behavioral assessments, but also eye‐tracking measurements (Onland‐Moret et al. 2020). Specifically, we tested whether breastfeeding duration was a good predictor of infant attentional disengagement around 5 and 10 months of age. These two measurement timepoints were both tested given the absence of prior evidence on the timing or trajectory of the hypothesized effect. Because language development has been shown to be sensitive to early‐life nutrition (Mahurin‐Smith 2015), we used child receptive vocabulary as a reference measure, to establish whether the positive effects of breastfeeding on child cognitive development previously reported were evident in the YOUth cohort.

## 2 Methods

### 2.1 Participants

The YOUth study is a large‐scale longitudinal cohort study with repeated measurements at regular intervals (“waves”). The study primarily focused on describing development of social competence and self‐regulation from a neurocognitive perspective, carrying out a broad range of measurements through a variety of research techniques. A description of the YOUth study rationale, design, and procedures can be found in Onland‐Moret et al. (2020). Measurements from four waves of the YOUth Baby & Child cohort (*N* = 2647) are used in the current study: breastfeeding report and attentional disengagement from the 5‐month wave and the 10‐month wave, and receptive vocabulary from the 3‐year wave and the 6‐year wave (see Table 1). Measurement waves were conducted within flexible age windows: the target age range was 4–6 months for the 5‐month wave, 9–11 months for the 10‐month wave, 2–4 years for the 3‐year wave, and 5–7 years for the 6‐year wave (Onland‐Moret et al. 2020). Median infant age at the time of eye‐tracking assessments was 23 weeks (IQR = 5 weeks) for the 5‐month wave and 45 weeks (IQR = 6 weeks) for the 10‐month wave. Median child age at the time of PPVT assessments was 189 weeks (IQR = 72 weeks) for the 3‐year wave and 315 weeks (IQR = 42 weeks) for the 6‐year wave. Demographic, birth‐related and maternal anthropometric data were collected through self‐report questionnaires. Participants were recruited between July 2015 and October 2023 in the province of Utrecht, including its major city Utrecht and a few cities on its borders. Relative to the Netherlands as a whole, this region is densely populated and comprises both urban and rural areas, all with good access to health care and related services. The eye‐tracking assessment using the gap‐overlap paradigm was scheduled to take around 15 minutes and the receptive vocabulary assessment was scheduled to take around 10 minutes (for details, see Onland‐Moret et al. 2020). The study was conducted in compliance with recognized standards for experimentation with human subjects. Approval was granted by the Medical Research Ethics Committee of the University Medical Center Utrecht and written informed consent was obtained for all participants.

**TABLE 1:** YOUth measurements included in the current study per wave.

Construct	Operationalization	YOUth baby & child			
		5 months	10 months	3 years	6 years
Breastfeeding duration	Age of the child (in months) at cessation of any breastfeeding	X	X		
Attentional disengagement	Gap‐effect derived from gap‐overlap paradigm	X	X		
Receptive vocabulary	Language quotient based on the PPVT‐3			X	X

*Note:* The target age range was 4–6 months for the 5‐month wave, 9–11 months for the 10‐month wave, 2–4 years for the 3‐year wave, and 5–7 years for the 6‐year wave (Onland‐Moret et al. 2020). Breastfeeding duration was based on short‐term parental recall in the 5‐ and 10‐month wave; when breastfeeding was ongoing, the reported value reflects the minimum known duration. A full overview of the collected data during each wave can be found on the Utrecht University website (YOUth Cohort Study, n.d.).

Infants known to have been born before 34 weeks' gestation (early and moderate preterm) and/or weighing under 2000 g were excluded due to their increased risk of atypical development (Pascal et al. 2018) and because they typically require specialized nutritional management. Data processing and exclusion steps per measure are described in the corresponding subsections (i.e., Section 2.2 Attentional disengagement, Section 2.3 Receptive vocabulary and Section 2.4 Breastfeeding duration). Because our sample sizes are based on data availability, no target sample size was calculated in advance for this study. For comparison, previous early‐life nutrition studies that utilized eye‐tracking in populations at risk of neurodevelopmental delay used samples ranging from 100 to 200 infants total (e.g., Leppänen et al. 2022; McCann et al. 2023). We exceed these numbers (all analyses of eye‐tracking measures were based on samples of > 1000 participants). To our knowledge, no comparable investigations (i.e., in a well‐nourished population) have been published previously.

**FIGURE 1:**
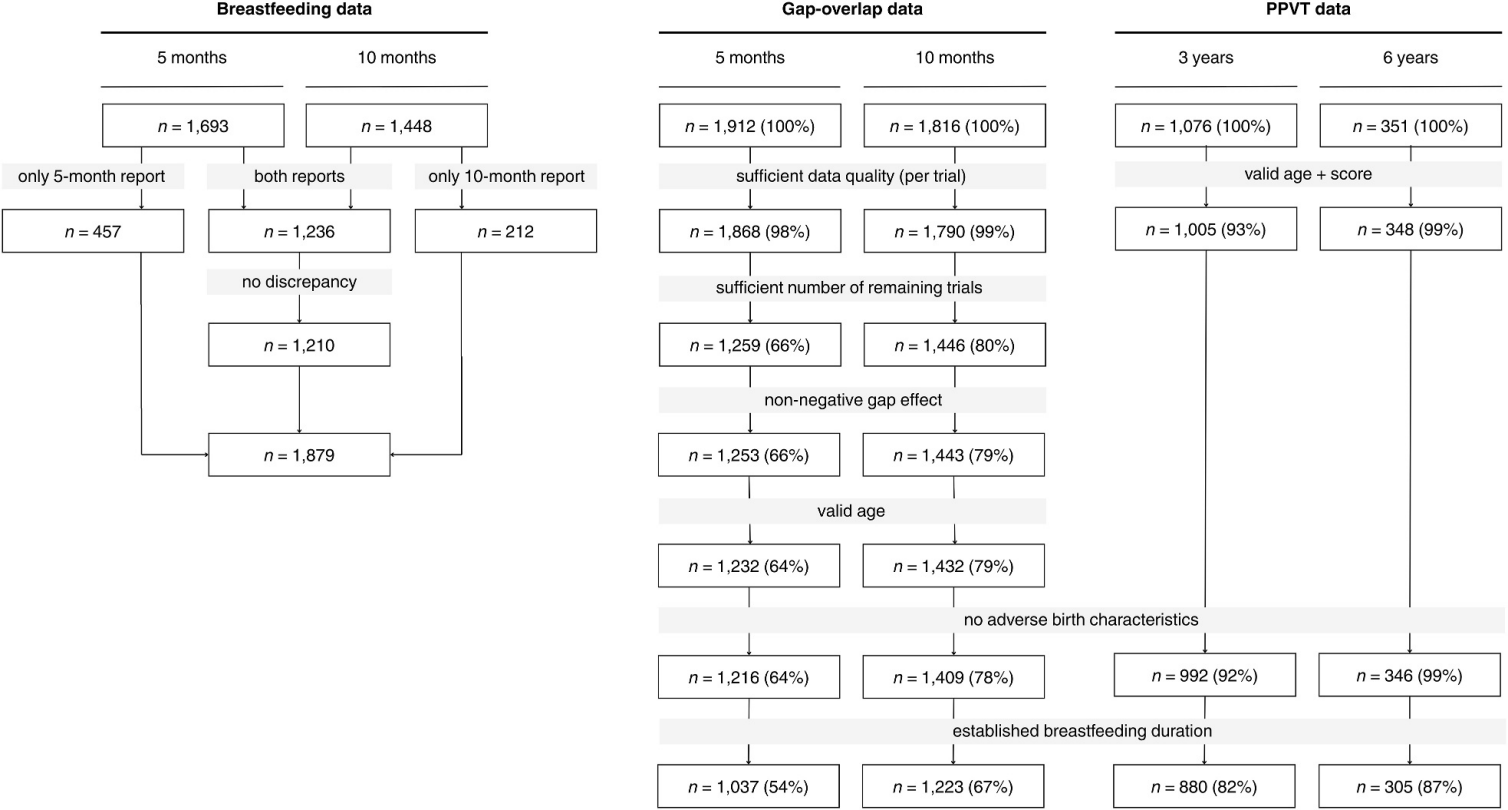
Participant flow diagram. The target age range was 4–6 months for the 5‐month wave, 9–11 months for the 10‐month wave, 2–4 years for the 3‐year wave, and 5–7 years for the 6‐year wave (Onland‐Moret et al. 2020). Inclusion criteria (seen in the gray boxes) were applied as specified in the main text. Exclusion of infants with adverse birth characteristics (i.e., < 34 weeks' gestation and/or birth weight < 2000 g) was performed at the penultimate step only for pragmatic reasons related to established data flows.

The sample of all participants included in at least one of the analyses (*n* = 1795) consisted of generally healthy infants. Mean birth weight was 3.5 kg, mean maternal age at the time of birth was 32 years and mean breastfeeding duration was 5 months. Note that breastfeeding duration was based on short‐term parental recall in the 5‐ and 10‐month wave; when breastfeeding was ongoing, the reported value reflects the minimum known duration (for details, see Section 2.4 Breastfeeding duration). Furthermore, most mothers were highly educated, and most households had a high monthly income (see Table 2). Participant characteristics were comparable across all samples we use for analysis (for the participant flow diagram, see Figure 1).

**TABLE 2:** Participant characteristics.

	*n*	Mean	SD
Days born before/after due date	1795	−0.44	9.75
Gestational age (weeks)	1795	39.94	1.39
Birthweight (grams)	1690	3531.68	471.60
Breastfeeding duration (months)	1795	5.28	3.32
Maternal age at the time of birth (years)	1795	32.46	3.68
Maternal pre‐pregnancy weight (kilograms)	1737	69.12	12.32
Maternal length (centimeters)	1738	170.98	6.54

	*n*	%
Sex		
Female	882	49.14
Male	913	50.86
Method of delivery		
Vaginal	1285	71.59
Vaginal, assisted delivery	144	8.02
Caesarean section	250	13.93
Not reported	116	6.46
Maternal highest completed level of education		
No completed education	2	0.11
Primary education	7	0.39
Secondary (vocational) education	227	12.65
Higher vocational education	496	27.63
Academic education	1006	56.04
Not reported	57	3.18
Monthly gross household income		
< €1250	9	0.50
€1250–€2000	22	1.23
€2000–€3000	107	5.96
€3000–€4000	275	15.32
> €4000	1253	69.81
Not reported	129	7.19
Breastfeeding duration (months)		
0 months	176	9.81
1 month	121	6.74
2 months	120	6.69
3 months	139	7.74
4 months	202	11.25
5 months	176	9.81
6 months	280	15.60
7 months	101	5.63
8 months	89	4.96
9 months	133	7.41
10 months	151	8.41
11 months	83	4.62
12 months	18	1.00
13 months	3	0.17
14 months	3	0.17

*Note:* Breastfeeding duration was based on short‐term parental recall in the 5‐ and 10‐month wave; when breastfeeding was ongoing, the reported value reflects the minimum known duration. Demographic and maternal anthropometric data were collected during pregnancy (around 20 weeks gestation) and birth‐related data were collected around birth.

### 2.2 Attentional Disengagement

#### 2.2.1 Apparatus

Infant gaze was recorded using the Tobii Pro TX300 eye tracker (Tobii Technology, Stockholm, Sweden) with an integrated 23‐inch monitor (1920 by 1080 pixels) and the Tobii Pro Spectrum with an integrated 23.8‐inch monitor (1920 by 1080 pixels), running at 300 and 600 Hz respectively. The latter proved to deliver gaze position signals of higher data quality (De Kloe et al. 2022) and therefore replaced the TX300 sometime into the project. The eye tracker communicated via the Tobii SDK controlled through MATLAB with PsychToolbox version 3 (Brainard 1997) used for stimulus presentation.

#### 2.2.2 Gap‐Overlap Paradigm

A more detailed description of the setup, procedure and stimuli can be found in de Zwart et al. (2025). In brief, infants were placed in a car seat at a set distance to the eye tracker screen, after which a 5‐point operator‐controlled calibration procedure was carried out. To evaluate attentional disengagement, the gap‐overlap paradigm was adapted from Elsabbagh et al. (2013). Conditions differ in the timing of the central stimulus offset relative to the peripheral stimulus onset: 200 ms in advance (gap), simultaneously (baseline), or the central stimulus remains on screen (overlap). Only gap and overlap conditions were considered for the present study, since the difference between these has been previously proposed as a reliable measure of attentional disengagement in infants (Cousijn et al. 2017).

All trials began with animated presentation of the central stimulus. When gaze was on the central stimulus, a random uniform interstimulus interval (ISI) of 600–700 ms followed. The next 200 ms comprised of either no stimulus on screen (gap condition) or a static central stimulus (overlap condition). Consequently, the peripheral stimulus appeared either to the left or the right of the central fixation point in a counterbalanced manner and remained displayed until the infant looked at it or until 2000 ms elapsed. Placement of the peripheral stimulus was at an eccentricity of approximately 20.8° from the center (1° = approximately 1.08 cm on the screen assuming participants are at 62 cm from the screen). Trials were presented in random order until either 16 trials were completed (per condition) or the infant refused further cooperation. If, upon completion, an insufficient number of trials were marked as successful based on a conservative online gaze‐contingent check, an additional four trials per condition were presented.

#### 2.2.3 Pre‐Processing of Eye‐Tracking Data

To estimate the gap effect, the eye‐tracking data were processed as follows. Fixations were classified using the identification by two‐means clustering (I2MC) algorithm v2.0, built specifically to deal with data across a wide range of noise levels and when periods of data loss may occur, as expected in infant eye‐tracking (Hessels et al. 2017). Default parameters were used. Each fixation was subsequently assigned to one of three areas of interest (AOIs): central stimulus, left peripheral stimulus or right peripheral stimulus. AOIs were constructed by evenly dividing the screen into three parts along its width, because for sparse stimuli large AOIs are most robust to noise (Hessels et al. 2016).

Crucially, SRT in the current study is defined as the time between onset of the peripheral stimulus and offset of the last fixation on center that is followed by peripheral fixation (in the left or right AOI), thus eliminating the influence of possible variations in saccade duration. If more than one such occurrence existed, the first saccade away from the center determined the trial SRT value. SRT were analyzed for successful trials only. To qualify as successful, (1) *any* peripheral fixation following center fixation had to be on the correct side of the screen (i.e., corresponding to the side where the peripheral stimulus had appeared)—though usually only one such occurrence takes place during a trial—and (2) trial SRT duration had to fall within the range of 100 to 2000 ms. Shorter SRTs were deemed anticipatory and longer SRTs were deemed excessively delayed. For each infant that produced at least 4 successful trials in a condition, median SRT (mdSRT) in that condition was calculated. Finally, the gap effect was computed by subtracting the gap mdSRT from the overlap mdSRT. As the gap effect decreases in size with age, a smaller gap effect is taken to represent a more mature response.

Notably, fixation classification and determining saccadic reaction time require eye‐tracking data of at least a certain quality. Infants often produce eye‐tracking data of lower quality as compared to older age groups (Hessels and Hooge 2019). This consideration is particularly relevant in studies that include multiple age groups, as differences in data quality can cause the appearance of differences in gaze behavior that in reality do not exist (Wass et al. 2014). We therefore operationalized the eye‐tracking data quality as precision (or variable error) and data loss. Accuracy (or systematic error) was not computed for this study specifically because it is not considered a potential issue for the present outcome measures, and is reported overall for the gap‐overlap paradigm in the YOUth cohort study by De Kloe et al. (2022). Precision is defined as the sample‐to‐sample reproducibility of a gaze position signal, assuming a stable gaze position. This was operationalized as the root mean square (RMS) of inter‐sample distances of the gaze position signal for each eye, then averaged across the two eyes. The I2MC algorithm provides robust classification of fixations up to 2° of RMS noise (Hessels et al. 2017), dictating exclusion of trials with RMS values greater than 2°. Data loss was calculated as the proportion of samples during which no gaze coordinate was reported relative to the sampling frequency of the eye tracker. Proportion of data loss was averaged over left and right eye per trial. Trials were excluded if data loss proportion exceeded 0.75, meaning less than 25% of the trial contained eye‐tracking data. If infants were more than 5 months older than the target age, or if age information was missing, their eye‐tracking records were excluded from analysis entirely.

Table 3 provides eye‐tracking data quality estimates per eye‐tracker and measurement wave, averaged over all trials. Table 4 reports the total number of trials and the number of trials not satisfying each of the data quality requirements per measurement wave.

**TABLE 3:** Eye‐tracking data quality prior to exclusion.

		5 months		10 months	
		Median	IQR	Median	IQR
RMS	TX300	1.18°	0.77°	0.81°	0.69°
	Spectrum	0.11°	0.04°	0.11°	0.04°
Data loss proportion	TX300	0.31	0.53	0.18	0.53
	Spectrum	0.07	0.27	0.04	0.28

*Note:* The target age range was 4–6 months for the 5‐month wave and 9–11 months for the 10‐month wave (Onland‐Moret et al. 2020). Precision was operationalized as the root mean square (RMS) sample‐to‐sample deviation and data loss as the proportion of a trial without valid gaze samples, averaged over all trials per eye‐tracker and measurement wave.

**TABLE 4:** Total number of trials and number of trials with insufficient data quality per wave.

	5 months		10 months	
	*n*	%	*n*	%
Trial numbers				
Total	70,060	100.00	62,224	100.00
RMS > 2°	6838	9.76	3156	5.07
Data loss proportion > 0.75	8370	11.95	7628	12.26

*Note:* The target age range was 4–6 months for the 5‐month wave and 9–11 months for the 10‐month wave (Onland‐Moret et al. 2020).

### 2.3 Receptive Vocabulary

A computerized version of the Dutch Peabody Picture Vocabulary Test, third edition (PPVT 3‐NL; Dunn and Dunn 2005; translated by Schlichting) was administered by trained research assistants. A description of the setup, procedure and stimuli can be found online in the Utrecht University archive (Junge, n.d.). In brief, each trial consisted of simultaneous presentation of a (pre‐recorded) spoken word and four pictures (one of which matched the word) on a 23.5‐inch screen. Two practice trials were conducted to begin with, in which the child was encouraged to touch or point to the matching word. Words become increasingly more complex over the course of the task. The PPVT 3‐NL has 204 words, divided in 17 sets of 12 items each. Each participant starts with an age‐appropriate set and continues until they make nine or more errors in one set. The raw score is calculated as: maximum score (12 × final set) minus the total number of errors. Raw scores were subsequently converted into age‐adjusted language quotient using the provided norms, which are based on 3‐month intervals (range: 2; 3–15; 11 years). Higher scores reflect better receptive vocabulary.

In the following cases, raw scores could not be converted into age‐adjusted language quotient according to the norms and were thus disregarded: any children who had completed the test at an age younger than 2 years and 3 months and any children who achieved a raw score that fell outside of the possible range dictated by the norms.

### 2.4 Breastfeeding Duration

The study design did not presuppose that infants would be breastfed, nor was breastfeeding continuation an inclusion criterion. Participating families were asked to report the infant's age (in months) at which they stopped any breastfeeding in the 5‐month wave and in the 10‐month wave (see Table 1). For “Never breastfed” the duration was set to 0 and for “Not yet stopped breastfeeding” the duration was set to the age of the child at the time of report, the latter reflecting the minimum known duration when breastfeeding had not been ceased.

For all other outcomes, breastfeeding duration was simply set to the reported age. The report in the 10‐month wave was used if available, otherwise substituted by report in the 5‐month wave. If both reports were available and a discrepancy of 2 months or more existed between them (e.g., parents report having stopped breastfeeding their child at 4 months of age when asked in the 5‐month wave, but at 6 months of age when asked in the 10‐month wave) the observation was deemed unreliable and excluded. No distinction was made between exclusive breastfeeding and mixed feeding.

Breastfeeding duration up to the infant's age at the eye‐tracking measurement in the 5‐month wave was used to predict gap‐overlap outcome measures in the 5‐month wave. To predict all other outcome measures, breastfeeding duration up to the infant's age at eye‐tracking measurement in the 10‐month wave was used.

### 2.5 Covariates

Our aim was to adjust for potential confounders, that is, variables that influence both the exposure and the outcome. Balancing the goal of approximating a minimally sufficient confounder set with practical constraints related to data availability and missingness within the cohort, we prioritized variables with the strongest and most consistently supported evidence as confounders in prior literature. Based on this approach, we selected the following variables: days born before/after due date, maternal age at the time of birth, maternal education, and household income (e.g., Pereyra‐Elías et al. 2022; Walfisch et al. 2013).

Importantly, age at the time of eye‐tracking was included as a predictor specifically for the gap‐overlap outcome measures (gap effect, mdSRT in gap condition and mdSRT in overlap condition). Age at the time of testing was not included as a predictor for the PVVT outcome, because the language quotient is already age‐adjusted based on the norms.

### 2.6 Statistical Analysis

We used multiple linear regression models to examine the association between breastfeeding duration and each outcome measure per wave. The gap effect models were first run with breastfeeding duration and age at the time of eye‐tracking as the only predictors. The PPVT language quotient models were first run with breastfeeding duration as the sole predictor. We conducted adjusted analyses by also including the potential confounders (i.e., days born before/after due date, maternal age at the time of birth, maternal education and household income level) for all models. We tested for linear and quadratic trends for maternal education and household income (the ordered categorical variables). The gap effect initially followed a right‐skewed distribution. A square root transformation was applied to achieve a more normal distribution. To allow for this transformation, infants who had negative gap effect values were excluded. We report unstandardized regression coefficients (*b*) for all predictors and standardized regression coefficients (*β*) for all numerical predictors.

As an exploratory analysis, we examined differences in gap effect and PPVT language quotient outcomes between infants breastfed for more than 4 months and those breastfed for 4 months or less. Group differences were tested using one‐tailed Mann–Whitney *U* tests, without adjustment for covariates. The 4‐month cut‐off was selected based on Dutch feeding guidelines, which recommend the introduction of complementary foods from 4 months onward, similarly to many other European countries (EFSA Panel on Dietetic Products and Nutrition and Allergies 2009). From this point, feeding patterns may begin to diversify. The hypothesized difference was that the group of infants breastfed for longer would perform better (i.e., lower gap effect and higher PPVT language quotient).

Furthermore, the association between infant attentional disengagement (gap effect) and child receptive vocabulary (PPVT language quotient) measures was assessed with Spearman's rank‐order correlation. The direction of the hypothesized association was negative, as we expected better performance in the gap‐overlap paradigm (i.e., lower gap effect) to correspond to better performance on the PPVT (i.e., higher PPVT language quotient).

We opted for complete‐case analysis because the study sample is relatively homogeneous (Fakkel et al. 2020; also see Table 2) and given the large sample size, loss of power is not a concern. Statistical analyses were conducted using R, version 4.4.2 (R Core Team 2023) and tested at a significance level of *α* = 0.05.

## 3 Results

### 3.1 Descriptive Statistics of the Gap‐Overlap Paradigm

On average, 15 eye‐tracking trials per participant per wave remained after exclusion (for information on variability, see Table 5). A difference depending on the condition of the task is observable in the expected direction (where overlap trials were less often successful and had longer saccadic reaction times). Within the same condition, participants in the 10‐month wave showed faster saccadic reaction times than participants in the 5‐month wave. Gap effect of both waves was similar. Descriptive statistics of gap‐overlap parameters as well as included and successful trial counts are provided in Table 5.

**TABLE 5:** Descriptive statistics of the gap‐overlap paradigm.

	5 months		10 months	
	Mean	SD	Mean	SD
Parameters				
mdSRT gap (ms)	326.02	73.80	251.05	35.92
mdSRT overlap (ms)	631.93	262.09	544.68	227.87
Gap effect	312.80	249.72	296.37	223.49
Trial counts				
Included trials gap	15.01	5.73	14.70	4.88
Included trials overlap	15.15	5.66	14.52	5.00
Successful trials gap	9.62	5.14	10.91	4.84
Successful trials overlap	6.15	4.02	8.15	4.09

*Note:* The target age range was 4–6 months for the 5‐month wave and 9–11 months for the 10‐month wave (Onland‐Moret et al. 2020). Parameters are reported only for infants who met the minimum required number of successful trials (as specified in the main text); values are otherwise untransformed.

Abbreviation: mdSRT, median saccadic reaction time.

### 3.2 Association Between Breastfeeding Duration and Infant Attentional Disengagement/Child Receptive Vocabulary

**FIGURE 2:**
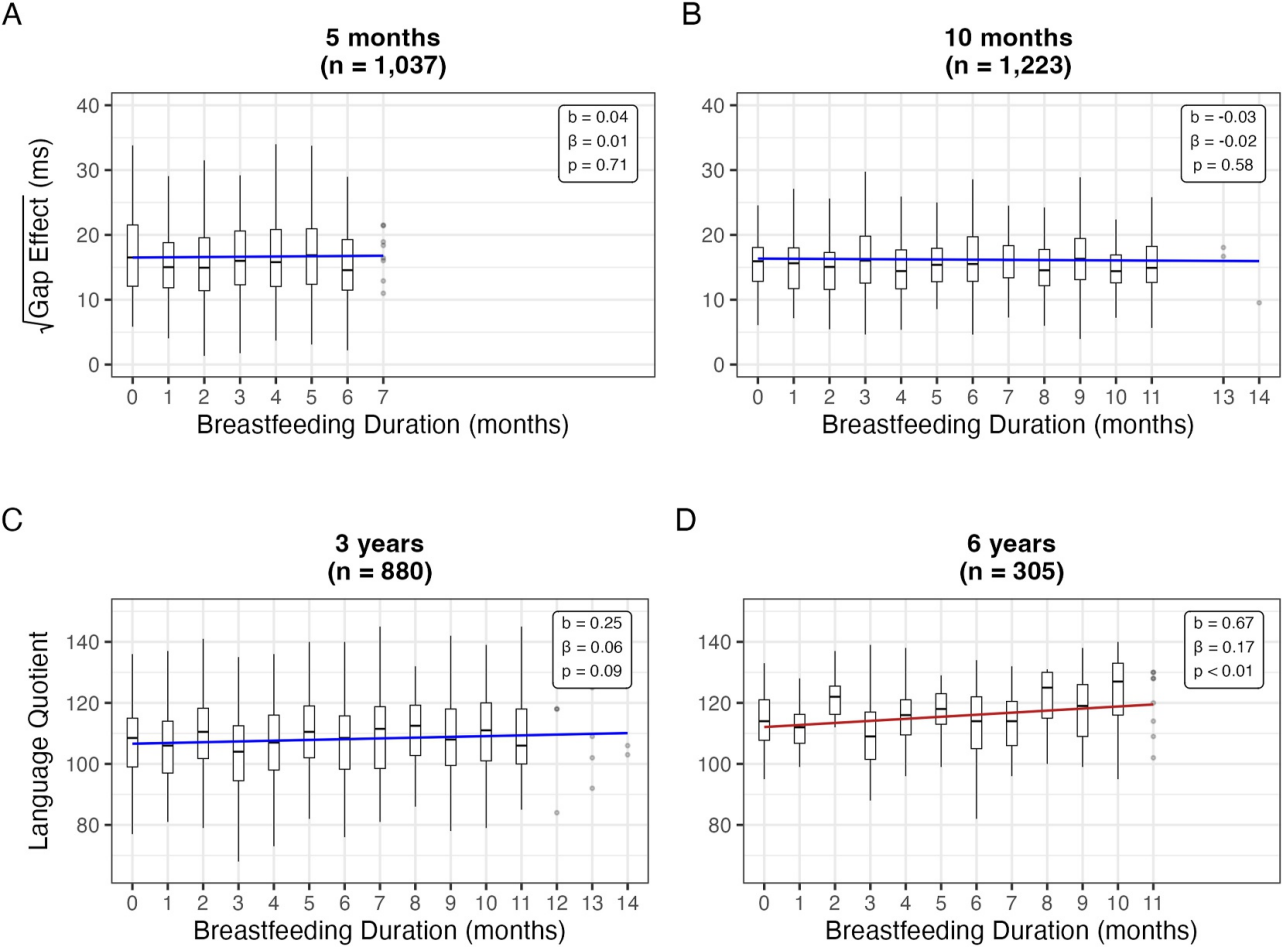
Simple linear regression models describing the bivariate association between breastfeeding duration and each outcome measure per wave. Panels A and B show square root‐transformed gap effect at 5 and 10 months, respectively; panels C and D show PPVT language quotient at 3 and 6 years, respectively. The target age range was 4–6 months for the 5‐month wave, 9–11 months for the 10‐month wave, 2–4 years for the 3‐year wave, and 5–7 years for the 6‐year wave (Onland‐Moret et al. 2020). For groups of < 10 participants, individual datapoints are displayed instead of boxplots. Red line color indicates a statistically significant association. Graphs were made using the R packages ggplot2, version 3.5.1 (Wickham 2011) and patchwork, version 1.3.2 (Pedersen 2025).

For visualization purposes, Figure 2 presents simple linear regression models showing the bivariate association between breastfeeding duration and each outcome measure per wave. An overview of the predictors used in the initial and adjusted linear regression models is given in Table 6.

**TABLE 6:** Predictors included in the linear regression models.

Predictor	Values
Breastfeeding duration	Numerical value between 0 and 14, see Section 2.4 *Breastfeeding duration* for details.
Age at the time of eye‐tracking	Numerical value in weeks.
Days born before/after due date	Numerical value in days. Negative if born before due date, positive if born after due date.
Maternal age at the time of birth	Numerical value in years.
Maternal education level	1 = No completed education; 2 = Primary education; 3 = Secondary (vocational) education; 4 = Higher vocational education; 5 = Academic education.
Household income level	1 = < €1250; 2 = €1250–€2000; 3 = €2000–€3000; 4 = €3000–€4000; 5 = > €4000.

*Note:* Breastfeeding duration was based on short‐term parental recall in the 5‐ and 10‐month wave; when breastfeeding was ongoing, the reported value reflects the minimum known duration.

Controlling for age at the time of eye‐tracking, breastfeeding duration was not found to significantly predict square root‐transformed gap effect (in the 5‐month wave: *b* = 0.10, *p* = 0.36, *n* = 1037; in the 10‐month wave: *b* = −0.03, *p* = 0.40, *n* = 1223). Omission of age at the time of eye‐tracking in the visualized gap effect models resulted in slightly different estimates that nonetheless support the same overall conclusion as seen in Figure 2 panel A and B (in the 5‐month wave: *b* = 0.04, *p* = 0.71, *n* = 1037; in the 10‐month wave: *b* = −0.03, *p* = 0.58, *n* = 1223).

In addition, median saccadic reaction time (mdSRT) in gap and overlap conditions were tested as outcome measures separately. Controlling for age at the time of eye‐tracking, breastfeeding duration did not significantly predict either gap mdSRT (in the 5‐month wave: *b* = 0.39, *p* = 0.72, *n* = 1320; in the 10‐month wave: *b* = 0.16, *p* = 0.59, *n* = 1357) or overlap mdSRT (in the 5‐month wave: *b* = 4.60, *p* = 0.29, *n* = 1,099, in the 10‐month wave: *b* = −0.98, *p* = 0.62, *n* = 1252).

While the predictive effect of breastfeeding duration on PPVT language quotient was not statistically significant in the 3‐year wave as seen in Figure 2 panel C (*b* = 0.25, *p* = 0.09, *n* = 880), the predictive effect of breastfeeding duration on PPVT language quotient was statistically significant in the 6‐year wave as seen in Figure 2 panel D (*b* = 0.67, *p* < 0.01, *n* = 305). The unstandardized regression coefficient *b* here may be interpreted as follows: for every month increase in breastfeeding duration, the associated PPVT language quotient increases by approximately 0.7 points. However, the observed variability in test scores among infants was high.

For all previously described models, adjusted versions including the previously specified potential confounders (see also Table 6) were estimated; these yielded no changes in the conclusions (see Tables 7 and 8). Diagnostic plots for the models are provided in (Supporting Information S1: Figures S1–S8).

**TABLE 7:** Estimates from regression models predicting square root‐transformed gap effect per wave.

Outcome	Square root‐transformed gap effect (5 months)						Square root‐transformed gap effect (10 months)					
Predictors	Initial model			Adjusted model			Initial model			Adjusted model		
(Intercept)	*b* = 19.28	NA	(*p* < 0.01)	*b* = 23.22	NA	(*p* < 0.01)	*b* = 19.29	NA	(*p* < 0.01)	*b* = 13.68	NA	(*p* < 0.01)
Breastfeeding duration	*b* = 0.10	*β* = 0.03	(*p* = 0.36)	*b* = 0.10	*β* = 0.03	(*p* = 0.40)	*b* = −0.02	*β* = −0.01	(*p* = 0.63)	*b* = −0.01	*β* = −0.01	(*p* = 0.82)
Age at the time of eye‐tracking	*b* = −0.13	*β* = −0.07	(*p* = 0.02)	*b* = −0.12	*β* = −0.07	(*p* = 0.05)	*b* = −0.07	*β* = −0.05	(*p* = 0.10)	*b* = −0.07	*β* = −0.05	(*p* = 0.08)
Days born before/after due date				*b* = −0.01	*β* = −0.02	(*p* = 0.52)				*b* = 0.01	*β* = 0.01	(*p* = 0.67)
Maternal age at the time of birth				*b* = −0.09	*β* = −0.05	(*p* = 0.10)				*b* = −0.13	*β* = −0.08	(*p* < 0.01)
Maternal education level (linear)				*b* = −2.79		(*p* = 0.42)				*b* = 4.81		(*p* = 0.05)
Maternal education level (quadratic)				*b* = 0.36		(*p* = 0.39)				*b* = −0.60		(*p* = 0.05)
Household income level (linear)				*b* = 2.23		(*p* = 0.33)				*b* = 1.32		(*p* = 0.43)
Household income level (quadratic)				*b* = −0.29		(*p* = 0.32)				*b* = −0.24		(*p* = 0.27)
Full model	*F* (2,1034) = 2.70 (*p* = 0.07)			*F* (8,956) = 1.16 (*p* = 0.32)			*F* (2,1220) = 1.51 (*p* = 0.22)			*F* (8,1125) = 2.84 (*p* < 0.01)		

*Note:* The target age range was 4–6 months for the 5‐month wave and 9–11 months for the 10‐month wave (Onland‐Moret et al. 2020).

**TABLE 8:** Estimates from regression models predicting PPVT language quotient per wave.

Outcome	PPVT language quotient (3 years)						PPVT language quotient (6 years)					
Predictors	Initial model			Adjusted model			Initial model			Adjusted model		
(Intercept)	*b* = 106.62	NA	(*p* < 0.01)	*b* = 90.63	NA	(*p* < 0.01)	*b* = 112.08	NA	(*p* < 0.01)	*b* = 111.02	NA	(*p* < 0.01)
Breastfeeding duration	*b* = 0.25	*β* = 0.06	(*p* = 0.09)	*b* = 0.19	*β* = 0.04	(*p* = 0.22)	*b* = 0.67	*β* = 0.17	(*p* < 0.01)	*b* = 0.64	*β* = 0.17	(*p* < 0.01)
Days born before/after due date				*b* = −0.01	*β* = −0.01	(*p* = 0.78)				*b* = −0.19	*β* = −0.15	(*p* = 0.01)
Maternal age at the time of birth				*b* = 0.03	*β* = 0.01	(*p* = 0.14)				*b* = −0.11	*β* = −0.03	(*p* = 0.58)
Maternal education level (linear)				*b* = 3.22		(*p* = 0.72)				*b* = −5.65		(*p* = 0.66)
Maternal education level (quadratic)				*b* = −0.20		(*p* = 0.86)				*b* = 0.87		(*p* = 0.57)
Household income level (linear)				*b* = 2.25		(*p* = 0.63)				*b* = 3.98		(*p* = 0.54)
Household income level (quadratic)				*b* = −0.23		(*p* = 0.71)				*b* = −0.23		(*p* = 0.78)
Full model	*F* (1,878) = 2.93 (*p* = 0.09)			*F* (7,813) = 1.42 (*p* = 0.19)			*F* (1,303) = 9.35 (*p* < 0.01)			*F* (7,265) = 3.57 (*p* < 0.01)		

*Note:* The target age range was 2–4 years for the 3‐year wave and 5–7 years for the 6‐year wave (Onland‐Moret et al. 2020).

When infants breastfed for over 4 months were compared to those breastfed for 4 months or less using one‐tailed Mann‐Whitney *U* tests, similar but slightly different outcomes were observed. Gap effect did not differ between the groups for either measurement wave while the PPVT language quotient was significantly higher in the group with longer breastfeeding duration for both measurement waves, as seen in (Supporting Information S1: Results S1).

#### 3.2.1 Repeated Analyses

Since each model was based on a different sample, we repeated analyses in subsets of the previous samples, using participants who had complete data for both PPVT language quotient in the 6‐year wave (which was found to be significantly predicted by breastfeeding duration) and square root‐transformed gap effect in the 5‐month wave or in the 10‐month wave (neither of which were found to be significantly predicted by breastfeeding duration). This was done to check if the different findings could be attributed to the use of different samples.

In the subset of participants who had complete data for both PPVT language quotient in the 6‐year wave and square root‐transformed gap effect in the 5‐month wave, breastfeeding duration was found to significantly predict PPVT language quotient in the 6‐year wave (*b* = 1.01, *p* < 0.01, *n* = 173). Controlling for age at the time of eye‐tracking, breastfeeding duration was not found to significantly predict square root‐transformed gap‐effect in the 5‐month wave (*b* = 0.43, *p* = 0.18, *n* = 173) in the same subset. In the subset of participants who had complete data for both PPVT language quotient in the 6‐year wave and square root‐transformed gap effect in the 10‐month wave, breastfeeding duration was found to significantly predict PPVT language quotient in the 6‐year wave (*b* = 0.87, *p* < 0.01, *n* = 244). Controlling for age at the time of eye‐tracking, breastfeeding duration was not found to significantly predict square root‐transformed gap‐effect in the 10‐month wave (*b* = −0.01, *p* = 0.94, *n* = 244) in the same subset. Again, adjusted versions of these models yielded no changes in the conclusions.

### 3.3 Association Between Infant Attentional Disengagement and Child Receptive Vocabulary

No association between the gap effect and PPVT language quotient was observed, as reported in the (Supporting Information S1: Results S2).

## 4 Discussion

Breastfeeding duration is typically associated with improved neurocognitive outcomes in childhood. In the present study, we investigated whether the previously established positive associations between breastfeeding and neurocognitive development in healthy children may already be detectable in early infancy using the gap‐overlap paradigm. While breastfeeding duration was found to be a good predictor for child receptive vocabulary in the 6‐year wave, it did not predict infant attentional disengagement measured by eye‐tracking in the 5‐month wave or in the 10‐month wave.

We did not observe an association between breastfeeding duration and the gap effect. However, contrary to prior expectations and a previous report (Macrae et al. 2024) the gap effect appeared to remain relatively stable from 5 to 10 months. The gap effect was used as an operationalization of attentional disengagement efficiency, which reflects the ability to disengage visual attention from a stimulus and is considered a basic component of early attentional control. The developmental trajectory of attentional disengagement across infancy and childhood in the YOUth cohort dataset has been described using pre‐processing steps comparable to ones used in the current study: indeed, it was found that the gap effect follows a slower maturation trajectory than the saccadic reaction times in the gap and overlap condition separately (de Zwart et al. 2025). We took this to suggest that saccadic reaction times could reveal differences that might be obscured when only considering the gap effect. Therefore, we proceeded with analyses of median saccadic reaction time in gap and overlap condition separately. There was also no association between breastfeeding duration and median saccadic reaction times of gap and overlap condition. Thus, we did not find any evidence for an association between breastfeeding duration and measures of attentional development in this study.

Numerous studies using the PPVT, which assesses receptive vocabulary, have reported higher scores among children who were breastfed for longer durations compared to those who were formula fed (Mahurin‐Smith 2015). Consistent with this literature, we observed a positive association between breastfeeding duration and receptive vocabulary at the 6‐year wave. No comparable association was detected at the 3‐year wave, despite regression coefficients being directionally similar. One possible interpretation is that associations between early‐life nutrition and language outcomes become more apparent as children age and language skills consolidate, a pattern that has been described previously, including reports of increasing associations between breastfeeding and verbal cognitive scores between 5 and 7 years of age (Pereyra‐Elías et al. 2022). Alternatively, greater variation in age at assessment in the 3‐year wave compared to the 6‐year wave may have reduced sensitivity to detect associations at earlier ages.

In contrast, the exploratory one‐tailed Mann–Whitney *U* tests we conducted indicated that the group of infants breastfed for over 4 months had better receptive vocabulary than the group of infants breastfed for 4 months or less, not only in the 6‐year wave but also in the 3‐year wave. This discrepancy between categorical comparison and linear regression model outcomes could be explained by a non‐linear association between breastfeeding duration and child outcomes. Previous investigators have observed a dose‐response relation between breastfeeding duration and child cognitive outcomes specifically, characterized by a strong association for the first few months of breastfeeding which diminished for longer durations (Borra et al. 2012). Although we did not have data on exclusivity, exclusive breastfeeding is known to be an important predictor of child PPVT outcomes as well (Belfort et al. 2013), and it is plausible that children breastfed for over 4 months were more likely to also have received exclusive breastfeeding in line with WHO recommendations (World Health Organization and United Nations Children's Fund 2003).

While we established some of the previously reported associations between breastfeeding and cognitive development to be evident in the YOUth cohort, we did not find breastfeeding duration to be predictive of infant attentional disengagement. A logical interpretation of our findings to consider is that for well‐nourished infants an association between breastfeeding duration and performance in the gap‐overlap paradigm does not exist. The large sample size strengthens our confidence that the null results are unlikely to be attributable to insufficient statistical power. However, this interpretation appears to contrast other studies that have suggested development of attention in infants is sensitive to effects of early‐life nutrition. We consider a few possible explanations.

First, the methodologies used to evaluate attention (or a specific aspect of it) vary widely across studies. For example, breastfed infants were previously suggested to have advanced maturation of attentional networks at 6 months of age compared to infants fed soy‐based formula, based on EEG outcomes (Gilbreath et al. 2023). While EEG may offer higher sensitivity than eye‐tracking for detecting early neurodevelopmental differences, the study did not include any task‐based assessments or behavioral measures designed to evaluate attentional function. In another study, long‐chain polyunsaturated fatty acid (LCPUFA) supplementation modulated improved attention of formula‐fed infants between 4 and 9 months of age (Colombo et al. 2011) and improved child cognitive functions, including PPVT scores at 5 years of age (Colombo et al. 2013). Note that the control group received a formula lacking LCPUFA, which is nowadays recognized as an essential nutrient for infant brain development. Attention in this study was assessed via heart rate, reflecting how deeply infants engage with a stimulus, while we assessed attentional disengagement efficiency. Though the gap‐overlap paradigm demonstrates relatively low test‐retest reliability (*r* = 0.50) (Cousijn et al. 2017), few eye‐tracking paradigms are as well‐established or widely used in developmental research. Regardless of methodological concerns however it may still be possible that breastfeeding is not related to attentional disengagement as such but is related to other aspects of attention, such as sustained engagement. Another avenue for future research concerns the potential role of these different aspects of visual attention in language acquisition. Currently there is evidence that infants' gaze‐following abilities are associated with later receptive vocabulary (Çetinçelik et al. 2021). At the same time, attentional disengagement has also been proposed as a potential contributor to early language development (D’Souza et al. 2020; Russo et al. 2021). To provide additional insight, we examined the association between our measures of infant attentional disengagement and child receptive vocabulary. All correlation coefficients were close to zero and none were statistically significant, as reported in Supporting information (Supporting Information S1: Results S2). Thus, we found no evidence of an association between infant attentional disengagement and child receptive vocabulary in the current sample.

Second, the studied populations differed in terms of developmental conditions. This could influence the potential for observable effects of breastfeeding. Note that our sample consisted of typically well‐nourished infants, most of whom came from families with high socioeconomic status (SES), which is thought of as a favorable developmental condition (Bradley and Corwyn 2002). Delayed development of visual attention has been observed in term infants suffering from malnourishment (Leppänen et al. 2022), nutritional deficiencies such as iron deficiency (McCann et al. 2023) and in other populations with adversity such as preterm infants (Burstein et al. 2021). It may be that the effects of early‐life nutrition on attentional development are more pronounced in populations that are in some way at risk of delay. Indeed, preterm infants showed improved attention functions when exposed to higher LCPUFA supplementation during the first months after birth (Westerberg et al. 2011). Furthermore, MRI studies have shown that breastfeeding supports gray matter volume and functional connectivity in frontal and parietal brain regions, which are involved in the control of attentional disengagement, in preterm infants (Yang et al. 2025) but not in healthy term infants (Deoni et al. 2018). In line with our current findings, term infants showed improved overall myelination, accompanied by improved higher‐order cognitive functions in childhood (Deoni et al. 2018).

Together, our findings in context of previous research suggest that early nutritional influences on attentional development may only be detectable under specific conditions, such as in at‐risk populations, or when using different neurophysiological measures, such as EEG. Some limitations apply to our findings. We have relied on retrospectively reported breastfeeding duration, which is known to be prone to recall bias (Stewart et al. 2024). To investigate the severity of this potential issue we compared the 5‐month report and 10‐month report when both were available, which was the case for the majority of participants who had given any breastfeeding report. We found (and excluded) 26 out of 1236 participants (0.21%) with a discrepancy greater than one month between the reports, which we did not judge to be evidence of problematic recall bias.

Secondly, we have substituted the 5‐month breastfeeding report when the 10‐month breastfeeding report was missing. This choice was driven by the intention to retain as much data as possible but likely leads to underestimation of breastfeeding duration for some infants. To assess whether this choice had substantially influenced our results, we reanalyzed the data instead only including participants who had a 10‐month breastfeeding report, which did not lead to altered conclusions (see Supporting Information S1: Tables S1 and S2). The participant characteristics in the sample of all participants included in at least one of these analyses (see Supporting Information S1: Table S3) also did not differ substantially from those in the sample of all participants included in at least one of our main analyses (see Table 2). Similarly, using untruncated breastfeeding duration for the child outcomes (i.e., PPVT language quotient in the 3‐year wave and in the 6‐year wave) did not lead to altered conclusions (see Supporting Information S1: Table S4).

We did not distinguish between infants who were predominantly breastfed, mixed‐fed, or predominantly formula‐fed. This primarily reflects the absence of detailed feeding data, though we emphasize the broader challenge of categorizing infant feeding patterns that vary and evolve over time. A lack of granularity in the breastfeeding exposure would be expected to attenuate associations. Though the operationalization of breastfeeding duration we employed is imperfect, the observed association with the PPVT language quotient leads us to conclude that it is adequate for the purposes of our study.

Regarding the outcome measures, the age ranges for each measurement wave need to be considered. While the PPVT language quotient was age‐adjusted according to the scoring manual, there exists no such standardized procedure for the gap effect. Instead, we corrected for age by including it as a predictor in the linear regression models. This correction is likely imperfect due to non‐linear development of attentional abilities. Although most infants were assessed within a relatively narrow 2‐month window around the target age, a portion of unexplained variance may be attributable to the variation in age.

We observed substantial attrition for the measures used in the present study. This was partly due to families deciding to quit study participation: reasons to quit may have included the required time commitment and shifting family priorities (e.g., due to the child's transition to school). However, a large part of the attrition was due to organizational constraints (e.g., limited time and budget) preventing follow‐up of all measurements for all children, rather than participant self‐selection. We did not find meaningful demographic differences between the samples of participants we used for analysis, providing us with no evidence of selective attrition.

Finally, given the observational nature of the data, causal interpretations should be made with caution. Despite adjusting for a number of potential confounders, residual confounding likely persists through factors left unaccounted for, most notably maternal cognitive ability.

### 4.1 Conclusion

We observed a positive association between breastfeeding duration and child receptive vocabulary. Despite establishing these differences in childhood, we did not observe an association between breastfeeding duration and infant attentional disengagement. Thus, while breastfeeding duration is associated with measurable differences in the cognitive development of well‐nourished infants at a later age, we conclude based on our findings that this is not the case for attentional development assessed through eye‐tracking at an earlier age. Future research could (a) aim to improve measurement techniques and paradigms for reliable assessment of attentional development at the individual level, and (b) explore populations with greater developmental variability. Finally, as feeding patterns vary and evolve over time, it remains imperative to carefully consider how breastfeeding exposure is defined and measured (e.g., exclusivity, duration, and timing) to help clarify the nature and strength of associations with attentional and cognitive development.

## Author Contributions

**Feline A. van Gelderen:** data curation, formal analysis, investigation, visualization, writing – original draft, methodology. **Roy S. Hessels:** data curation, investigation, methodology, writing – review and editing, supervision. **Kelly A. Mulder:** conceptualization, methodology, supervision, writing – review and editing. **Lidewij Schipper:** conceptualization, methodology, project administration, writing – review and editing, supervision.

## Ethics Statement

The study was conducted in compliance with recognized standards for experimentation with human subjects. Approval was granted by the Medical Research Ethics Committee of the University Medical Center Utrecht and written informed consent was obtained for all participants.

## Conflicts of Interest

The authors declare no conflicts of interest.

## Supporting information


Supporting Information S1

## Data Availability

Data can be formally requested from the YOUth cohort study website: https://www.uu.nl/en/research/youth‐cohort‐study/request‐youth‐data. The experimental code for the gap‐overlap paradigm is available via https://github.com/UtrechtUniversity/youth‐tasks‐matlab‐kkc/blob/master/saccade/saccade_task.m. The pre‐processing scripts for the YOUth eye‐tracking data are available via https://git.science.uu.nl/R.S.Hessels/YOUTH_ET_QC/.
