# Supplementary material for: The Association Between Breastfeeding Duration and Attentional and Cognitive Development in Infancy and Early Childhood
Source: Infancy. 2026 Aug 19;31(4):e70120. doi: 10.1111/infa.70120 (PMC13490886; doi:10.1111/infa.70120)
Supplement: Supplementary file 1 [file INFA-31-0-s001.docx]

**Supporting Information**

We used data collected as part of the broad-scope Youth Of Utrecht (YOUth) cohort study, which is described in Onland-Moret et al. (2020). Diagnostic plots for our main linear regression models (both basic and adjusted) are provided in Supporting Figures 1–4. Note that model assumptions were judged through visual assessment of diagnostic plots rather than tested formally, as it is known that such tests may provide false positives especially with large samples (Shatz, 2024). Deviations from normality were observed in the tails of some model residuals (see Supporting Figures 2, 5-8) however it has been shown that linear regression models without normally distributed errors are valid, given sufficient sample size (Knief & Forstmeier, 2021; Schmidt & Finan, 2018).

**Supporting Figure 1**. Model diagnostics plots for basic (panel A) and adjusted (panel B) linear regression models predicting square root-transformed gap effect in the 5-month wave.

B

A

**Supporting Figure 2**. Model diagnostics plots for basic (panel A) and adjusted (panel B) linear regression models predicting square-root transformed gap effect in the 10-month wave.

B

A

**Supporting Figure 3**. Model diagnostics plots for basic (panel A) and adjusted (panel B) linear regression models predicting PPVT language quotient around 3 years.

B

A

A

**Supporting Figure 4**. Model diagnostics plots for basic (panel A) and adjusted (panel B) linear regression models predicting PPVT language quotient around 6 years.

B

A

**Supporting Figure 5**. Model diagnostics plots for basic (panel A) and adjusted (panel B) linear regression models predicting gap mdSRT around 5 months.

**Supporting Figure 6**. Model diagnostics plots for basic (panel A) and adjusted (panel B) linear regression models predicting gap mdSRT around 10 months

B

A

**Supporting Figure 7**. Model diagnostics plots for basic (panel A) and adjusted (panel B) linear regression models predicting gap mdSRT around 5 months.

B

A

**Supporting Figure 8**. Model diagnostics plots for basic (panel A) and adjusted (panel B) linear regression models predicting overlap mdSRT around 10 months

B

A

**Supporting Results 1**

One-tailed Mann-Whitney U tests were used to compare infants breastfed for over 4 months to those breastfed for 4 months or less. The hypothesized difference was that the group of infants breastfed for longer would perform better (i.e., lower gap effect and higher PPVT language quotient). The results indicated that there was no significant difference between these groups in terms of gap effect around 5 months (U = 135807, p = 0.64) and around 10 months (U = 172746, p = 0.90), and that the group of infants breastfed for over 4 months had significantly higher PPVT language quotient around 3 years (U = 83594, p = 0.01) and around 6 years (U = 9432.5, p-value < 0.01).

**Supporting Results 2**

Spearman’s rank-order correlation was used to assess the association between the gap effect (our measure of infant attentional disengagement) and the PPVT language quotient (our measure of child receptive vocabulary). We hypothesized that the association would be negative, as we expected better performance in the gap-overlap paradigm (i.e., lower gap effect) to correspond to better performance on the PPVT (i.e., higher PPVT language quotient). No significant associations were observed between the gap effect and PPVT language quotient. Specifically, the gap effect around 5 months was not significantly associated with PPVT language quotient around 3 years (r_s_ = 0.04, n = 524, p = 0.79) or around 6 years (r_s_ = 0.05, n = 177, p = 0.76). Likewise, the gap effect around 10 months was not significantly associated with PPVT language quotient around 3 years (r_s_ = 0.02, n = 672, p = 0.71) or around 6 years (r_s_ = -0.01, n = 245, p = 0.41).

**Supporting Table 1**

*Estimates from regression models predicting square root-transformed gap effect per wave, in the sample of infants who had a breastfeeding report around 10 months*

|  | **Outcome** | **Square root-transformed gap effect (5 months)** | | | | | | | **Square root-transformed gap effect (10 months)** | | | | | |
| --- | --- | --- | --- | --- | --- | --- | --- | --- | --- | --- | --- | --- | --- | --- |
| **Predictors** | | *Initial model* | | | | *Adjusted model* | | | *Initial model* | | | *Adjusted model* | | |
| (Intercept) | | b = 19.82 | NA | (p < 0.01) | | b = 25.32 | NA | (p < 0.01) | b = 19.50 | NA | (p < 0.01) | b = 16.76 | NA | (p = 0.01) |
| Breastfeeding duration | | b = 0.03 | β = 0.01 | (p = 0.81) | | b = 0.05 | β = 0.01 | (p = 0.74) | b = -0.02 | β = -0.01 | (p = 0.72) | b = -0.02 | β = -0.01 | (p = 0.71) |
| Age at the time of eye tracking | | b = -0.14 | β = -0.08 | (p = 0.04) | | b = -0.13 | β = -0.07 | (p = 0.08) | b = -0.07 | β = -0.05 | (p = 0.11) | b = -0.07 | β = -0.05 | (p = 0.10) |
| Days born before/after due date | |  |  |  | | b = -0.02 | β = -0.03 | (p = 0.47) |  |  |  | b = 0.02 | β = 0.04 | (p = 0.28) |
| Maternal age at the time of birth | |  |  |  | | b = -0.12 | β = -0.06 | (p = 0.09) |  |  |  | b = -0.15 | β = -0.08 | (p < 0.01) |
| Maternal education level (linear) | |  |  |  | | b = -3.42 |  | (p = 0.38) |  |  |  | b = 4.78 |  | (p = 0.07) |
| Maternal education level (quadratic) | |  |  |  | | b = 0.44 |  | (p = 0.36) |  |  |  | b = -0.58 |  | (p = 0.07) |
| Household income level (linear) | |  |  |  | | b = 2.55 |  | (p = 0.41) |  |  |  | b = -0.11 |  | (p = 0.96) |
| Household income level (quadratic) | |  |  |  | | b = -0.35 |  | (p = 0.37) |  |  |  | b = -0.06 |  | (p = 0.81) |
| **Full Model** | | F(2,754) = 2.18 (p = 0.11) | | | F(8,704) = 1.07 (p = 0.38) | | |  | F(2,1012) = 1.38 (p = 0.25) | | | F(8,940) = 2.59 (p < 0.01) | | |

*Note*. The target age range was 4–6 months for the 5-month wave and 9–11 months for the 10-month wave (Onland-Moret et al., 2020).

**Supporting Table 2**

*Estimates from regression models predicting PPVT language quotient per wave, in the sample of infants who had a breastfeeding report around 10 months*

|  | **Outcome** | **PPVT language quotient (3 years)** | | | | | | | **PPVT language quotient (6 years)** | | | | | |
| --- | --- | --- | --- | --- | --- | --- | --- | --- | --- | --- | --- | --- | --- | --- |
| **Predictors** | | *Initial model* | | | | *Adjusted model* | | | *Initial model* | | | *Adjusted model* | | |
| (Intercept) | | b = 106.80 | NA | (p < 0.01) | | b = 89.66 | NA | (p < 0.01) | b = 111.94 | NA | (p < 0.01) | b = 114.53 | NA | (p < 0.01) |
| Breastfeeding duration | | b = 0.24 | β = 0.06 | (p = 0.12) | | b = 0.15 | β = 0.04 | (p = 0.35) | b = 0.72 | β = 0.19 | (p < 0.01) | b = 0.67 | β = 0.18 | (p < 0.01) |
| Days born before/after due date | |  |  |  | | b = -0.02 | β = -0.01 | (p = 0.78) |  |  |  | b = -0.18 | β = -0.13 | (p = 0.04) |
| Maternal age at the time of birth | |  |  |  | | b = -0.00 | β = -0.00 | (p = 0.98) |  |  |  | b = -0.04 | β = -0.01 | (p = 0.88) |
| Maternal education level (linear) | |  |  |  | | b = 5.80 |  | (p = 0.55) |  |  |  | b = -4.68 |  | (p = 0.74) |
| Maternal education level (quadratic) | |  |  |  | | b = -0.46 |  | (p = 0.70) |  |  |  | b = 0.79 |  | (p = 0.64) |
| Household income level (linear) | |  |  |  | | b = 0.54 |  | (p = 0.92) |  |  |  | b = -0.72 |  | (p = 0.93) |
| Household income level (quadratic) | |  |  |  | | b = -0.04 |  | (p = 0.95) |  |  |  | b = 0.35 |  | (p = 0.74) |
| **Full Model** | | F(1,731) = 2.38 (p = 0.12) | | | F(7,681) = 1.38 (p = 0.21) | | |  | F(1,258) = 9.32 (p < 0.01) | | | F(7,226) = 3.00 (p < 0.01) | | |

*Note*. The target age range was 2–4 years for the 3-year wave and 5–7 years for the 6-year wave (Onland-Moret et al., 2020).

**Supporting Table 3**

*Participant characteristics, in the sample of infants who had a breastfeeding report around 10 months*

|  | *n* | Mean | | SD |
| --- | --- | --- | --- | --- |
| **Days born before/after due date** | 1,363 | -0.44 | | 9.80 |
| **Gestational age** (weeks) | 1,363 | 39.94 | | 1.40 |
| **Birthweight** (grams) | 1,298 | 3,533.23 | | 474.86 |
| **Breastfeeding duration** (months) | 1,363 | 5.73 | | 3.53 |
| **Maternal age at the time of birth** (years) | 1,363 | 32.42 | | 3.64 |
| **Maternal pre-pregnancy** **weight** (kilograms) | 1,329 | 68.96 | | 12.03 |
| **Maternal length** (centimeters) | 1,329 | 171.06 | | 6.55 |
|  | *N* | | % | |
| **Sex** |  | |  | |
| Female | 681 | | 49.96 | |
| Male | 682 | | 50.04 | |
| **Method of delivery** |  | |  | |
| Vaginal | 999 | | 73.29 | |
| Vaginal, assisted delivery | 108 | | 7.92 | |
| Caesarean section | 185 | | 13.57 | |
| Not reported | 71 | | 5.21 | |
| **Maternal highest completed level of education** |  | |  | |
| No completed education | 2 | | 0.15 | |
| Primary education | 6 | | 0.44 | |
| Secondary (vocational) education | 159 | | 11.67 | |
| Higher vocational education | 382 | | 28.03 | |
| Academic education | 780 | | 57.23 | |
| Not reported | 34 | | 2.49 | |
| **Monthly gross household income** |  | |  | |
| <€1,250 | 6 | | 0.44 | |
| €1,250-€2,000 | 14 | | 1.03 | |
| €2,000-€3,000 | 77 | | 5.65 | |
| €3,000-€4,000 | 211 | | 15.48 | |
| >€4,000 | 967 | | 70.95 | |
| Not reported | 88 | | 6.46 | |
| **Breastfeeding duration** (months) |  | |  | |
| 0 months | 134 | | 9.83 | |
| 1 month | 92 | | 6.75 | |
| 2 months | 89 | | 6.53 | |
| 3 months | 105 | | 7.70 | |
| 4 months | 95 | | 6.97 | |
| 5 months | 68 | | 4.99 | |
| 6 months | 223 | | 16.36 | |
| 7 months | 80 | | 5.87 | |
| 8 months | 86 | | 6.31 | |
| 9 months | 133 | | 9.76 | |
| 10 months | 151 | | 11.08 | |
| 11 months | 83 | | 6.09 | |
| 12 months | 18 | | 1.32 | |
| 13 months | 3 | | 0.22 | |
| 14 months | 3 | | 0.22 | |

*Note*. Breastfeeding duration was based on short-term parental recall in the 10-month wave; when breastfeeding was ongoing, the reported value reflects the minimum known duration. Information displayed in the table was accumulated across different measurement waves: demographic and maternal anthropometric data were collected during pregnancy (around 20 weeks gestation) and birth-related data were collected around birth.

**Supporting Table 4**

*Estimates from regression models predicting PPVT language quotient per wave, based on untruncated breastfeeding duration*

|  | **Outcome** | **PPVT language quotient (3 years)** | | | | | | | **PPVT language quotient (6 years)** | | | | | |  |
| --- | --- | --- | --- | --- | --- | --- | --- | --- | --- | --- | --- | --- | --- | --- | --- |
| **Predictors** | | *Initial model* | | | | *Adjusted model* | | | *Initial model* | | | *Adjusted model* | | |  |
| (Intercept) | | b = 106.55 | NA | (p < 0.01) | | b = 90.50 | NA | (p < 0.01) | b = 112.10 | NA | (p < 0.01) | b = 110.47 | NA | (p < 0.01) |  |
| Breastfeeding duration | | b = 0.26 | β = 0.06 | (p = 0.07) | | b = 0.20 | β = 0.05 | (p = 0.17) | b = 0.66 | β = 0.18 | (p < 0.01) | b = 0.63 | β = 0.18 | (p < 0.01) |  |
| Days born before/after due date | |  |  |  | | b = -0.01 | β = -0.01 | (p = 0.78) |  |  |  | b = -0.19 | β = -0.15 | (p = 0.01) |  |
| Maternal age at the time of birth | |  |  |  | | b = 0.03 | β = 0.01 | (p = 0.84) |  |  |  | b = -0.11 | β = -0.03 | (p = 0.59) |  |
| Maternal education level (linear) | |  |  |  | | b = 3.22 |  | (p = 0.72) |  |  |  | b = -5.56 |  | (p = 0.66) |  |
| Maternal education level (quadratic) | |  |  |  | | b = -0.20 |  | (p = 0.86) |  |  |  | b = 0.85 |  | (p = 0.58) |  |
| Household income level (linear) | |  |  |  | | b = 2.24 |  | (p = 0.63) |  |  |  | b = 4.19 |  | (p = 0.52) |  |
| Household income level (quadratic) | |  |  |  | | b = -0.23 |  | (p = 0.71) |  |  |  | b = -0.26 |  | (p = 0.76) |  |
| **Full Model** | | F(1,878) = 3.29 (p = 0.07) | | | F(7,813) = 1.47 (p = 0.18) | | |  | F(1,303) = 9.61 (p < 0.01) | | | F(7,265) = 3.61 (p < 0.01) | | |  |

*Note*. The target age range was 2–4 years for the 3-year wave and 5–7 years for the 6-year wave (Onland-Moret et al., 2020).

**References**

Knief, U., & Forstmeier, W. (2021). Violating the normality assumption may be the lesser of two evils. *Behavior Research Methods*, *53*(6), 2576–2590. https://doi.org/10.3758/s13428-021-01587-5

Onland-Moret, N. C., Buizer-Voskamp, J. E., Albers, M. E. W. A., Brouwer, R. M., Buimer, E. E. L., Hessels, R. S., de Heus, R., Huijding, J., Junge, C. M. M., Mandl, R. C. W., Pas, P., Vink, M., van der Wal, J. J. M., Hulshoff Pol, H. E., & Kemner, C. (2020). The YOUth study: Rationale, design, and study procedures. *Developmental Cognitive Neuroscience*, *46*, 100868. https://doi.org/10.1016/j.dcn.2020.100868

Schmidt, A. F., & Finan, C. (2018). Linear regression and the normality assumption. *Journal of Clinical Epidemiology*, *98*, 146–151. https://doi.org/10.1016/j.jclinepi.2017.12.006

Shatz, I. (2024). Assumption-checking rather than (just) testing: The importance of visualization and effect size in statistical diagnostics. *Behavior Research Methods*, *56*(2), 826–845. https://doi.org/10.3758/s13428-023-02072-x
